#  Isolated Jejunal Duplication Cyst Associated with Intestinal Malrotation in a Newborn

**DOI:** 10.21699/jns.v5i4.455

**Published:** 2016-10-10

**Authors:** Prasanta Kumar Tripathy, Pradeep Kumar Jena, Hiranya Kishor Mohanty

**Affiliations:** Department of Pediatric Surgery, SVP PG Institute of Pediatrics, Cuttack, Odisha, India

**Dear Sir**

Alimentary tract duplications are epithelial-lined cystic or tubular structures attached to bowel wall and supplied by mesenteric vessels. Association between intestinal malrotation and duplication cyst, especially of jejunal location is rarely mentioned in literature[1-5]. We are reporting a rare coexistence of isolated jejunal duplication cyst and malrotation in a neonate with intestinal obstruction.

A 15-day-old male neonate was brought to emergency for multiple episodes of bilious vomiting. The baby was born full-term by spontaneous vaginal delivery at home and antenatal ultrasonography(USG) was not done. At presentation, he was 2500 gm, moderately dehydrated, pulse 160/min and respiratory rate was 46/min. Resuscitation was started immediately and the nasogastric tube inserted. Abdomen was soft, non-tender and a mobile mass was palpable in left upper abdomen. Plain X-ray abdomen showed distended stomach and duodenum. Rest of the abdomen was gasless, but there was presence of rectal gas in pelvis (Fig.1). USG revealed a well defined thick walled cystic lesion in left mid abdomen measuring 42х37mm suggestive of duplication cyst (Fig.1). Doppler imaging showed "Whirlpool sign" in epigastric region with superior mesenteric vein (SMV) curving from right to left of superior mesenteric artery (SMA) suggesting midgut malrotation (Fig.1). At surgery, a non-communicating jejunal duplication cyst, intimately adherent to jejunal wall and compressing the intestinal lumen was found (Fig.1). Malrotation was also present which was corrected. Duplication cyst was excised along with a portion of adjoining jejunum. End-to-end jejunal anastomosis was done. Postoperative period was uneventful. 

**Figure:**
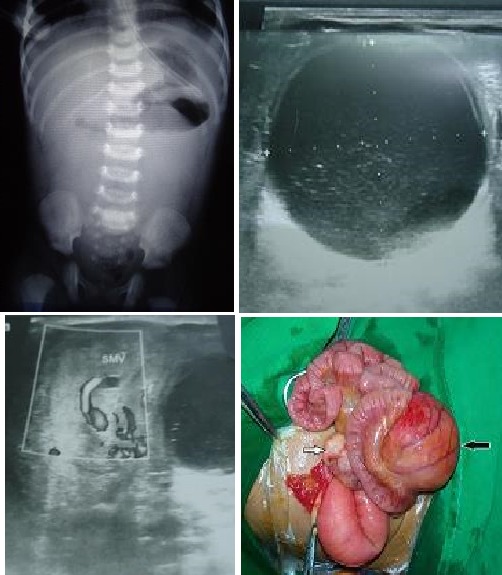
Figure 1: Showing plain x-ray, ultrasound abdomen, Doppler USG, and operative picture of jejunal duplication cyst (black arrow).The sub-hepatic mobile cecum and appendix suggested midgut malrotation (white arrow).

Combination of enteric duplication and intestinal malrotation is a rare entity and may present as an acute surgical emergency [1-5]. Gastric and duodenal distension on plain X-ray of abdomen is suggestive of duodenal obstruction. The presence of rectal gas in addition to dilated stomach and duodenum will commonly point towards partial duodenal obstruction. This can occur in malrotation due to compression of distal duodenum by Ladd's bands [3]. Similarly our preoperative suspicion was malrotation on plain X-ray abdomen. Color Doppler imaging showing Whirlpool flow pattern of SMV around SMA is suggestive of midgut malrotation. Ultrasonography is very reliable in diagnosing duplication cysts.It will show an inner hyperechoic rim of mucosa-submucosa and an outer hypoechoic rim of muscular layer [2]. Duplication cysts are thick walled because it is composed of smooth muscle and mucosa as opposed to other types of cysts such as mesenteric and omental cysts. Our preoperative diagnosis coincided with operative findings.Surgical removal of duplication cyst preserving normal bowel is possible in some cases, but is extremely difficult [1]. Resection of duplication with the native bowel is the usual procedure due to common blood supply [3, 5]. This case represents a rare coexistence of two congenital malformations of GIT. Ultrasonography is very helpful in detecting both duplication cyst and malrotation.

## Footnotes

**Source of Support:** Nil

**Conflict of Interest:** None
